# Prevalence and incidence of cyclic vomiting syndrome in Japan: A study using Japanese claims data

**DOI:** 10.1371/journal.pone.0279502

**Published:** 2022-12-22

**Authors:** Chia-Hsien Chang, Toshiyuki Hikita, Nobuyoshi Takabayashi, Motonobu Sakaguchi

**Affiliations:** 1 Takeda Pharmaceutical Company Limited, Osaka, Japan; 2 Hikita Pediatric Clinic, Gumma, Japan; University of South Carolina, UNITED STATES

## Abstract

Cyclic vomiting syndrome (CVS) is a gastrointestinal disorder that is characterized by recurrent episodes of vomiting. Previous studies have provided reliable data on the prevalence of CVS among children in Japan; however, neither prevalence data nor incidence of CVS is available for adults. Hence, we obtained detailed prevalence and incidence data for CVS and estimated the total number of CVS cases in Japan. This retrospective cross-sectional study was conducted using the JMDC (JMDC, Inc.; formerly known as Japan Medical Data Center Co., Ltd.,) database, which is a de-identified Japanese claims database. Individuals enrolled between January 2017 and December 2017 were included in this study. Longitudinal data for eligible populations were used to identify patients who displayed CVS symptoms throughout the follow-up period. Due to the lack of a specific diagnosis code for CVS in 2017, the Rome IV criteria were applied to identify CVS cases in the pediatric and adult populations. The prevalence was standardized to the 2017 Japanese census and extrapolated to estimate the number of CVS cases. A total of 2,093 patients with CVS were identified from 3,506,144 individuals. The overall age-and-sex-standardized prevalence was 0.32 per 1,000 population (95% confidence interval [CI]: 0.30–0.34), projected to approximately 49,000 patients with CVS across Japan. The pediatric age-and-sex-standardized prevalence was 2.10 per 1,000 population (95% CI: 2.01–2.19), and the adult prevalence was 0.05 per 1,000 population (95% CI: 0.04–0.06). Marked sex differences were observed before and after 12 years of age. Thus our study provides the first large-population-based estimates of CVS prevalence and incidence in Japan, and currently, the only estimates for adult CVS in Japan.

## Introduction

The prevalence of cyclic vomiting syndrome (CVS) has increased among adults in Western countries in addition to pediatric patients. CVS is a gut-brain interaction disorder characterized by recurrent, stereotypical episodes of severe nausea and vomiting interspersed with periods of little or no symptoms [1]. CVS was previously considered a pediatric disorder; however, recent data from English-speaking countries have indicated its prevalence in adults as well [2]. The etiology of CVS remains unclear; however, there may be a link between CVS and migraine since CVS is associated with a personal history of migraine and a favorable response to antimigraine therapy [3, 4]. Both pediatric and adult patients with CVS demonstrate several comorbidities [5, 6], poor quality of life [7], and suboptimal care [8].

Epidemiological data for CVS, report a prevalence of 1.9% among children aged 5–15 years in Scotland [9] and Fitzpatrick et al. estimated an incidence rate of 3.15 (95% confidence interval [CI]: 2.19–4.11) per 100,000 children in Ireland [10]. A recent review including 26 studies reported the prevalence of CVS in children and adolescents to be 0.2%–6.2% internationally [11]. Inconsistent prevalence rates reported across studies could be the result of different settings. Moreover, the incidence of CVS is rarely reported. In Japan, neither the prevalence nor the incidence of CVS in the adult population has been reported. The clinical and epidemiological features of CVS have not been studied well.

Acute nausea and vomiting are common symptoms and physicians usually recommend seeking medical care when the symptoms are persistent and severe. Hence, a healthcare claims database can be used to estimate the epidemiology of CVS, which may address any problems associated with recall and non-response rates often found in self-reported data. One study, using a US claims database, investigated the epidemiology of CVS when its diagnosis was made [12]. In Japan, there is a healthcare insurance claims database that includes all medical claims from outpatients, emergency departments, and inpatients, which is suitable for studying under-recognized conditions, such as CVS. Utilizing such an administrative source, we aimed to investigate the epidemiology of CVS in both pediatric and adult populations and to characterize the clinical features of CVS cases in Japan.

## Materials and methods

### Data sources

This retrospective cross-sectional study was performed using the Japanese JMDC (JMDC, Inc.; formerly known as Japan Medical Data Center Co., Ltd.,) claims database (JMDC). The JMDC, a large employee-based medical administrative health insurance claims database, contains longitudinal integrated medical and pharmaceutical claims. As of November 2018, over 5.9 million individuals were enrolled in the JMDC, representing approximately 5% of the total Japanese population. Data were collected from various databanks nationwide, including public and private insurance claims that capture de-identified individual medical information. Thus, the data were not linked to individual patient medical records.

### Study population

Since the base population is designated to be representative of the source population in 2017, all patients continuously enrolled between January 2017 through December 2017, except those born in 2017, were included in the study. Longitudinal data from January 2005 to September 2018 were used to identify unexplained vomiting episodes (VE) in the base population. To investigate the incidence, the population was limited to those who did not have an original onset of vomiting symptoms consistent with a potential CVS diagnosis prior to 2017.

An unexplained VE is defined as the occurrence of vomiting without any known etiology. Japanese patients tend to utilize the national healthcare system, and physicians tend to utilize all tests available as there is no financial limitation on doing so; therefore, vomiting episodes that have a known etiology tend to be associated with a particular condition. In medical claims databases, these conditions are identified as particular disease codes, and cases of unknown etiology lack such codes. Thus, by excluding all cases associated with a code, we were able to retain cases of unexplained VE, which may represent a potential patient pool of CVS cases.

### CVS case definition

Until May 2019, a unique disease code for CVS was not available in Japan. Since some physicians had not yet used the new disease code and CVS tended to be underdiagnosed, CVS cases in this study were identified using symptom-based criteria. Rome IV has established a uniform symptom-based framework that is applicable in clinical practice [13] and is the only criterion for diagnosing CVS in adults [14, 15]. The diagnostic criteria for CVS in children, as proposed by the North American Society for Pediatric Gastroenterology, Hepatology and Nutrition [16] and the International Headache Society [17], suggest two sets of cutoff values for vomiting frequency by time interval, and the severity of vomiting is assessed on an hourly basis. This claim database can determine the service received in time; however, such granularity of data cannot be retrieved from the datasets. In contrast, Rome IV is relatively simple and similar to the criteria for adults. Given that this study was designed to compare populations of children, adolescents, and adults, Rome IV was chosen for consistency.

Considering the patient’s age on the index date, which is defined as the first date when patients met CVS case requirements, a patient who sought medical help due to vomiting was considered to have prevalent CVS if one of the following conditions was met before or on December 31, 2017: (1) for children aged ≤ 12 years, if the patient had experienced ≥2 unexplained vomiting episodes in 6 months; (2) for adolescents aged 13–18 years, if the patient had experienced ≥2 unexplained vomiting episodes in 6 months; and (3) for adults aged ≥19 years, if the patient had experienced ≥2 unexplained vomiting episodes in 6 months and at least three discrete episodes in the previous year. Regarding the incidence rate, we considered only cases of potential CVS when the first occurrence of CVS was in 2017.

Patients were excluded if they had been diagnosed with predefined medical conditions within 30 days before and after the date of occurrence of vomiting. The patients who remained in the study experienced repeated vomiting with no known etiology. Furthermore, physicians may use some descriptors unique to Japanese claims data to flag patients with CVS [18]. This would have been the only way to identify patients with CVS before the code for CVS was introduced to Japanese claims data. Consequently, the number of patients who were flagged as potential CVS cases was used to help describe the perception of CVS in clinical practice. Hereafter, this practice is referred to as a flagging descriptor.

### Statistical methods

Comorbidities were identified based on the medical diagnoses presented within 180 days before and at the index date. The comorbid conditions were classified into common diseases [19–21] and corresponding symptoms/complications [19, 21] during a VE among the pediatric and adult populations. The use of migraine-related treatments was investigated since CVS has been associated with migraine headaches and abdominal migraine, using 30 days as an observation interval for better characterization of the symptoms or complications present when the event occurred. Disease characteristics were reported as categorical variables using 0 or 1 to represent either the absence or presence of the disease, respectively. Crude prevalence and incidence rates for different case definitions were age-and-sex-standardized to the population estimate in October 2017, based on Japanese national census data [22]. A Poisson distribution was used to calculate 95% CIs for prevalence and incidence, and gamma distribution was used for directly standardized rates since it is a weighted sum of independent Poisson random variables [23]. As a descriptive study, there are no *a priori* hypotheses; hence, no hypothesis testing was conducted.

### Ethical considerations

Since the data used for estimation are the number of CVS cases, the privacy and confidentiality of the data are well protected. Although patients can be identified via a unique identifier by the JMDC, the secondary database used for research purposes only has de-identified data; therefore, obtaining informed consent was waived. This protocol was approved by the ethical committees of the Research Institute of Healthcare Data Science with respect to scientific content and compliance with the applicable research guidelines (RI2019016).

## Results

### Case characteristics

**Fig 1:**
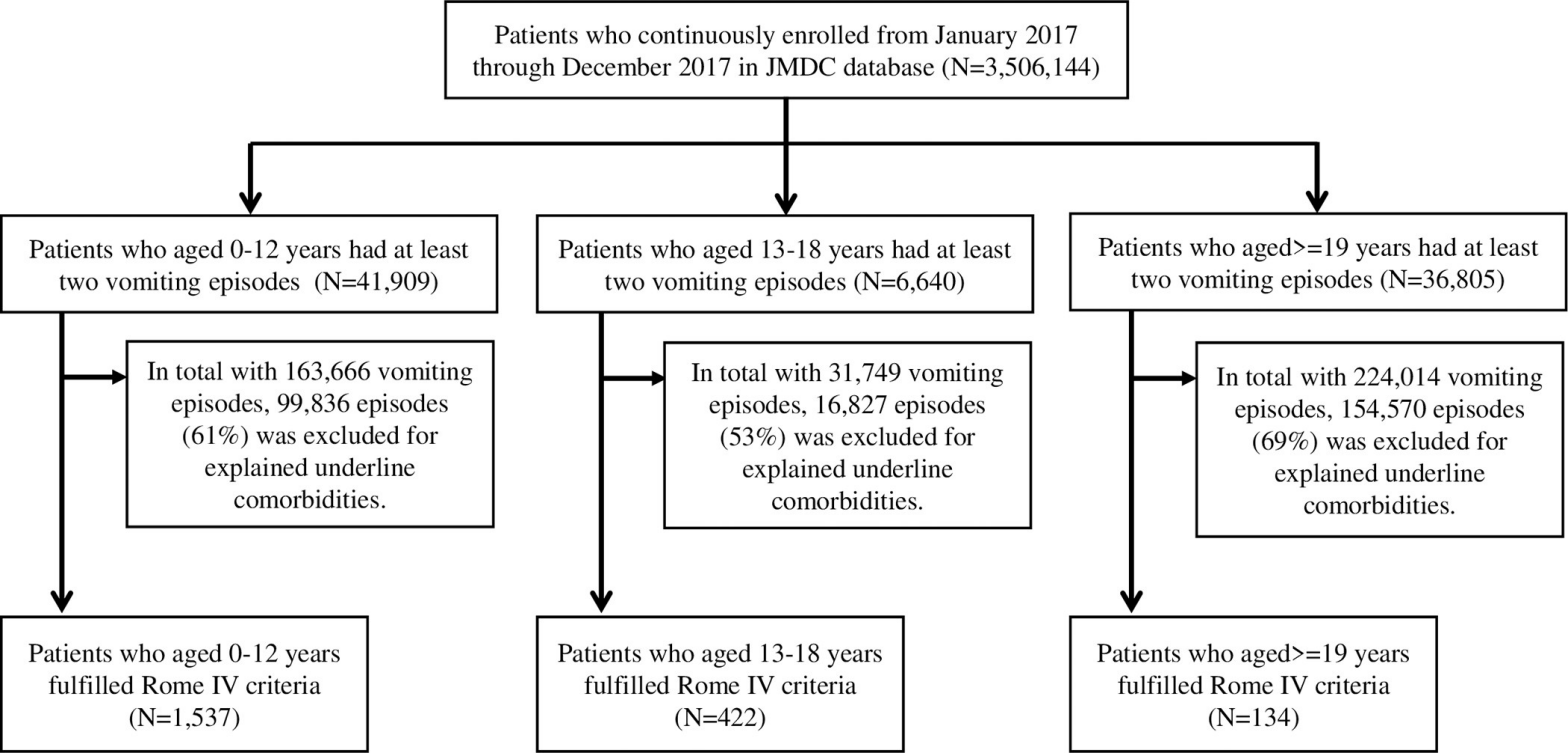
Flow chart of the study population and identified CVS cases.

In total, 2,093 CVS cases fulfilled the Rome IV criteria with regard to prevalence analysis, out of 3,506,144 individuals registered in the JMDC database on or before December 31, 2017, and 527 cases occurred in 2017. A composite of 19,458,436 person-years was eligible for incidence analysis (Fig 1).

Approximately 54% of the patients were children aged ≤12 years old, with a mean age of 5 years at the index date. The majority of children with CVS were males (57%). However, this trend shifted to a female majority among adolescents (53%). Furthermore, this trend was even more pronounced among adults, with female patients comprising 66% of the total adult population. We discovered that 36% of the children were flagged with a CVS descriptor; however, this trend decreased from 21% among adolescents to only 8% in adult patients.

Our study suggests that both children and adults are affected by CVS, although clinical presentation varies with age. Developmental disorders were the most common baseline diseases among children. At the time of case identification, the clinical presentation included diarrhea, dehydration, and hypoglycemia. Among those aged > 12 years, migraine, anxiety, and depression were the most common diseases at baseline. With increasing age, a decreasing trend of symptoms, such as diarrhea, dehydration, and hypoglycemia, was observed at the time of meeting the Rome IV criteria. However, an increasing trend in vertigo/dizziness and headache was also observed, especially among adolescents (Table 1).

**Table 1:** Characteristics of identified CVS cases.

Variables	Total numbers of patients with CVS (N = 2093)		
	Children (0–12 years, N = 1537)	Adolescents (13–18 years, N = 422)	Adults (19–74 years, N = 134)
Demographic variables			
Age at time of index date	Mean 5 (SD 3)	Mean 13 (SD 3)	Mean 32 (SD 17)
Female	660 (43%)	224 (53%)	89 (66%)
Have any flagging descriptor of CVS			
Never	979 (64%)	334 (79%)	123 (92%)
Any time after registering in JMDC	558 (36%)	88 (21%)	11 (8%)
Common morbidities at baseline			
Anxiety	14 (0.9%)	36 (8%)	21 (16%)
Migraine	24 (2%)	73 (17%)	33 (25%)
Developmental disorder	81 (5%)	28 (7%)	8 (6%)
Complex regional pain Syndrome	15 (1%)	22 (5%)	5 (4%)
Depression	6 (0.4%)	33 (8%)	22 (16%)
Dysautonomia	1 (0.1%)	9 (2%)	4 (3%)
Irritable bowel syndrome	9 (0.6%)	26 (6%)	8 (6%)
Event-time characteristic*			
Fever	47 (3%)	7 (2%)	1 (0.8%)
Diarrhea	299 (20%)	48 (11%)	3 (2%)
Headache	37 (2%)	58 (14%)	9 (7%)
Vertigo/dizziness	8 (0.5%)	14 (3%)	10 (8%)
Dehydration	180 (12%)	31 (7%)	7 (5%)
Hypoglycemia	56 (4%)	4 (1%)	2 (2%)

The data is represented as Mean (SD) for continuous data or as frequency (percentage) for categorical data.

CVS, cyclic vomiting syndrome; SD, standard deviation.

* The disease/condition that appeared at the time of meeting the Rome-IV criteria.

### Prevalence and incidence rate

During 2017, the overall age-and-sex-standardized prevalence was 0.32 per 1,000 population (95% CI: 0.30–0.34 per 1,000), projected to approximately 49,000 patients with CVS across Japan. Subgroup analyses of the prevalence by age and sex revealed interesting findings. The pediatric age-and-sex-standardized prevalence was 2.10 per 1,000 population (95% CI: 2.01–2.19 per 1,000), and the adult age-and-sex-standardized prevalence was 0.05 per 1,000 population (95% CI: 0.04–0.06 per 1,000). While the pediatric prevalence in females was similar to that in males, the adult prevalence in females was over twice that in males (0.08 [95% CI: 0.06–0.10] vs. 0.03 [95% CI: 0.02–0.06]) per 1,000 population (Table 2).

**Table 2:** Adjusted prevalence of patients with CVS by age and sex.

Age (years)	Male patients		Female patients		Total		
	Cases (N)	Proportion per 1,000 individuals (95% CI)	Cases (N)	Proportion per 1,000 individuals (95% CI)	Cases (N)	Proportion per 1,000 individuals (95% CI)	Expected patients with CVS across Japan
Children (0–12 years)	877	2.73 (2.56–2.92)	660	2.17 (2.01–2.34)	1537	2.46 (2.34–2.58)	32,845
Adolescent (13–18 years)	198	1.28 (1.11–1.48)	224	1.54 (1.34–1.75)	422	1.41 (1.28–1.55)	9,830
Pediatrics (0–18 years)	1075	2.24 (2.10–2.37)	884	1.95 (1.83–2.09)	1959	2.10 (2.01–2.19)	42,674
Adult (19–74 years)	45	0.03 (0.02–0.06)	89	0.08 (0.06–0.10)	134	0.05 (0.04–0.06)	6,475
Overall, age/sex-standardized to Japan population	1120	0.27 (0.25–0.29)	973	0.42 (0.39–0.45)	2093	0.32 (0.30–0.34)	49,149

CI, confidence interval; CVS, cyclic vomiting syndrome.

The standardized overall incidence rate of CVS was 1.83 per 100,000 person-years (95% CI 1.65–2.02 per 100,000), which is projected to be less than 2,800 new CVS cases per year. Although CVS may affect individuals of any age, its incidence rate by age most often occurs in individuals between 0 and 12 years of age. Approximately 2,000–2,500 children and adolescents in Japan fulfill the Rome IV criteria for CVS annually (Table 3).

**Table 3:** Adjusted incidence rate of patients with CVS by age and sex.

Age (years)	Male patients		Female patients		Total		
	Cases (N)	Rate per 100,000 person-years (95% CI)	Cases (N)	Rate per 100,000 person-years (95% CI)	Cases (N)	Rate per 100,000 person-years (95% CI)	Expected new patients with CVS per year
Children (0–12 years)	221	18.18 (15.48–21.18)	164	14.20 (11.88–16.83)	385	16.24 (14.44–18.20)	2,171
Adolescent (13–18 years)	44	4.61 (3.35–6.19)	70	7.67 (5.98–9.59)	114	6.10 (5.03–7.33)	426
Pediatrics (0–18 years)	265	13.52 (11.68–15.54)	234	11.97 (10.32–13.79)	499	12.76 (11.51–14.10)	2,596
Adult (19–74 years)	7	0.07 (0.03–0.20)	21	0.30 (0.18–0.51)	28	0.15 (0.10–0.24)	200
Overall, age/sex-standardized to Japan population	272	1.49 (1.29–1.72)	255	2.44 (2.12–2.80)	527	1.83 (1.65–2.02)	2,796

CI, confidence interval; CVS, cyclic vomiting syndrome.

### Management of CVS cases

There are a few recommended treatment plans for CVS. Following episodes of vomiting, prokinetic agents such as domperidone and metoclopramide were the most frequently used. While most of these medications are administered orally, approximately 5% of CVS cases receive parenteral administration, presumably due to recurrent vomiting which makes oral administration ineffective. In addition, antiemetics such as 5-HT_3_ antagonists are rarely used. Another mode of treatment may include intravenous fluids to provide nutritive or hydrating support to offset severe vomiting. Intravenous treatment was administered to 30% of the children, 18% of the adolescents, and 15% of the adults with CVS. Sedatives or pain relief medications are recommended in cases of pain. While the use of sedatives, such as short-acting benzodiazepines, was less frequent, a similar strategy for pain relief was observed across all ages (Table 4).

**Table 4:** Coadministration of identified CVS cases.

Variables	Total numbers of patients with CVS (N = 2093)		
	Children (0–12 years, N = 1537)	Adolescents (13–18 years, N = 422)	Adults (19–74 years, N = 134)
*Treatment for CVS*			
**Prokinetics**	**869 (57%)**	**241 (57%)**	**87 (65%)**
Domperidone	818 (53%)	214 (51%)	69 (51%)
Oral	460 (30%)	198 (47%)	65 (49%)
Rectal	490 (32%)	30 (7%)	7 (5%)
Metoclopramide	144 (9%)	45 (11%)	27 (20%)
Oral	54 (4%)	22 (5%)	20 (15%)
Parenteral	102 (7%)	25 (6%)	7 (5%)
**5-HT**_**3**_ **antagonist**	1 (0.1%)	-	
**Sedatives**	**81 (5%)**	**17 (4%)**	**15 (11%)**
Chlorpromazine	4 (0.3%)	3 (0.7%)	2 (2%)
Diazepam	42 (3%)	9 (2%)	8 (6%)
Lorazepam	-	3 (1%)	4 (3%)
Hydroxyzine	32 (2%)	2 (0%)	1 (1%)
Midazolam	11 (1%)	-	-
Promethazine	-	-	1 (1%)
**Supportive care**	**425 (30%)**	**76 (18%)**	**20 (15%)**
Electrolytes with carbohydrates	267 (18%)	46 (11%)	9 (7%)
Glucose	127 (8%)	15 (4%)	6 (5%)
Sodium chloride	245 (16%)	45 (11%)	9 (6%)

The data is represented as frequency (percentage) for categorical data.

CVS, cyclic vomiting syndrome.

### Use of migraine-related treatment

Abortive triptans were found to be prescribed as a treatment for 1% of children, 12% of adolescents, and 28% of adults. The most frequently used abortive therapies were rizatriptan and sumatriptan. Amitriptyline, a first-line prophylactic medication for migraine attacks, is the most frequently prescribed medication in adolescents and adults. The use of prophylactics to prevent migraine attacks was generally low, with the exception of cyproheptadine (Table 5).

**Table 5:** Use of migraine-related treatment among identified CVS cases.

Variables	Total numbers of patients with CVS (N = 2093)		
	Children (0–12 years, N = 1537)	Adolescents (13–18 years, N = 422)	Adults (19–74 years, N = 134)
*Abortive therapy for migraine*			
**Triptan**	**9 (1%)**	**50 (12%)**	**37 (28%)**
Dihydroergotamine	1 (0.1%)	5 (1%)	-
Ergotamine, combinations	-	1 (0.2%)	-
Sumatriptan	3 (0.2%)	5 (1%)	7 (5%)
Naratriptan	-	6 (1%)	6 (4%)
Zolmitriptan	1 (0.1%)	7 (2%)	5 (4%)
Rizatriptan	2 (0.1%)	17 (4%)	13 (10%)
Eletriptan	1 (0.1%)	10 (2%)	8 (6%)
*Prophylactic therapy for migraine*			
Amitriptyline	2 (0.1%)	10 (2%)	5 (4%)
Nortriptyline	-	1 (0.2%)	-
Phenobarbital	18 (1%)	7 (2%)	1 (0.8%)
Valproate sodium	25 (2%)	36 (9%)	18 (13%)
Topiramate	-	2 (0.5%)	-
Levetiracetam	9 (1%)	3 (0.8%)	-
Zonisamide	3 (0.2%)	1 (0.2%)	2 (2%)
Riboflavin	16 (1%)	5 (1%)	1 (1%)
Propranolol	-	2 (0.5%)	-
Mirtazapine	-	-	2 (1.5%)
Lomerizine hydrochloride	3 (0.2%)	16 (4%)	6 (5%)
Cyproheptadine	278 (18%)	25 (6%)	1 (0.8%)

The data is represented as frequency (percentage) for categorical data.

CVS, cyclic vomiting syndrome.

## Discussion

To the best of our knowledge, this is the first population-based epidemiological study to provide real-world evidence of the prevalence and incidence of CVS across all ages in Japan. Our data suggest that the age-and-sex-standardized prevalence of symptoms fulfilling the Rome IV criteria for CVS was 0.32 per 1,000 population in Japan in 2017 and that the standardized incidence rate was 1.83 per 100,000 person-years. Prevalence and incidence varied with age. The highest prevalence and incidence were observed in children, followed by adolescents and adults. Our findings suggest that the estimated prevalence of 2.10 per 1,000 people is similar to that reported in the Japanese pediatric population [24, 25] however, the estimated prevalence in Japanese subpopulations was lower than that reported in English-speaking countries, especially among adults.

The US-representative web-based survey using Rome symptom-based criteria estimated the prevalence of CVS as 1.1% (11 per 1,000 population) for children and adolescents (4–18 years) and 1.2% (12 per 1,000 population) for adults [26–28]. However, self-reported data for both children and adults cannot completely exclude organic medical causes since there was no subsequent physician confirmation for this type of investigation. Regarding studies on the incidence of the disease, one study estimated an incidence rate of 9.35 per 100,000 person-years overall, and 12.24 per 100,000 person-years in children, using a US claims database [12]. Yet another study suggested that there was no significant difference in CVS prevalence between racial or ethnic groups [26]; however, the conclusion remains controversial since the Asian population is under-represented. Furthermore, intrinsic and extrinsic factors may play a role in explaining variations in CVS. For instance, the prevalence of migraine headache, a periodic disorder similar to CVS, appears to be lower in the Asia-Pacific region than in Europe and North America, and the rates of migraine with aura differ substantially among Asian countries [29]. Since CVS and migraine are disorders involving neuronal hyperexcitability, the signaling pathways that trigger attacks may vary with ethnicity and lifestyle. For instance, differences in policies between countries regarding marijuana use, such as the recreational or medical use of cannabinoids in the US, could contribute to a variety of cannabis exposures. Interestingly, there seemed to be a higher prevalence of CVS cases after the legislation decriminalizing marijuana was passed in a given area [30, 31]. To date, no trigger factors for CVS have been reported in Asian populations, and further investigation is needed.

The willingness to seek medical attention for vomiting may be unique to Japan. Japanese citizens utilize healthcare facilities much more frequently than in other countries, such as the G7 [32]. Although Japan has an aging population, and the older adults are more likely to seek medical attention for disease management, the government works to raise awareness about disease prevention at the population level. While there may be some differences between subsidies among different socioeconomic groups within Japan, all citizens are covered by the national insurance system at a low cost, and the medical costs paid by Japanese citizens are lower than those in the US [33, 34]. For instance, children’s medical expenses are subsidized by local government copayment, and a medical-aid program exists for specific pediatric chronic diseases, including CVS [35]. Hence, parents are more likely to take children to a medical facility to follow up on the incidence of vomiting. Similarly, adults may also be willing to seek medical care for vomiting to prevent illness in the first place since it is a symptom that calls for attention [36]. Therefore, this data source can adequately capture the occurrences of vomiting that seek medical services.

The prevalence of CVS in Japan has been reported at 0.22, to be 0.23% in Japan, based on studies in pediatric populations [24, 25]. Our finding in pediatric CVS prevalence is comparable with previous studies conducted in Japan. While the prevalence of CVS in adults appears to be similar to that in children [2], our findings suggest that CVS occurs more commonly in children. We investigated the possibility of understanding how the threshold of unexplained vomiting episodes affects the prevalence of CVS in children and adults. When the criteria used for adults were applied to the children (i.e., adding the requirement of three discrete episodes in the past year), the calculated rate for the pediatric population significantly reduced from 2.10 to 0.13 per 1,000 individuals. The rate ratio of pediatric to adult patients was 2.6:1 when applying the same threshold, which was consistent with the primary result of a higher prevalence in the pediatric population than in adults. This test highlighted the potential drawback of using different cutoff times to characterize CVS, especially in the context of patient behavior. Further studies are required to explore the disparity between the prevalence of adult CVS found in this study and the general assumptions regarding the nature of CVS in adults.

The CVS cases identified in the JMDC database had demographic characteristics similar to those reported in Western countries. Both our study and a Scottish study showed that children with CVS had an average age of onset of around 5 years, and among younger children, males were more commonly affected than females [9]. Similarly, a summary of adult CVS comparisons reported that females were more affected than males, with a median age of 37 years [20]. While some countries may use 5-HT_3_ antagonists, such as ondansetron, which is used to treat nausea among chemotherapy patients, physicians usually do not prescribe this drug in Japan. Similarly, only a small proportion of the patients received parenteral administration of intravenous fluids or prophylactic medications. As 5-HT_3_ antagonists are not approved for CVS treatment in Japan, we were unable to accurately ascertain the use of this drug from medical claims data. In addition, the low percentage of patients who received prophylactic medications could be attributed to the fact that Japan does not have any guidelines regarding accepted treatment suggestions for patients with CVS. Moreover, due to the unavailability of Japanese practice guidelines, there was a lack of information related to comorbidities. In the present study, lower than expected rates of comorbidities such as migraine, anxiety, and irritable bowel syndrome were found. This could be attributed to the fact that the information was not collected and submitted systematically when analyzing healthcare claims data. Given that claims data may represent a clinical cost driver, severe or moderate symptoms associated with effective treatment are likely to have good records; however, mild symptoms without intervention may not be evident in terms of the available data found within healthcare records.

Owing to its perceived rarity, CVS within the adult population is usually diagnosed after ruling out other diseases. While adult patients with CVS can clearly describe their symptoms, which helps physicians to diagnose CVS more frequently, the data in the present study showed that only 8% of patients within the adult population have a flagging descriptor. The small proportion of adults with a flagging descriptor implied that physicians, in general, tend not to recognize CVS as a potential diagnosis in adults. Moreover, our data showed that the percentage of patients receiving prokinetics, sedatives, and support care, which are commonly used to treat CVS, would be consistent with a larger percentage than the proportion of patients with a CVS descriptor. This also indicates that CVS is under-diagnosed in adults [37]. Moreover, we found the possibility of a lack of CVS diagnosis among patients with associated ailments such as migraines, depression, and irritable bowel syndrome. Early diagnosis of CVS in the adult population can improve the patient’s condition and quality of life. However, due to the lack of awareness regarding CVS in the adult population, such patients may have been excluded without a diagnosis or a continuing treatment plan [8].

The strength of this population-based study is that it investigated the prevalence and incidence of CVS across all age groups in Japan. There have been some global studies related to prevalence among children, but no studies have addressed the incidence rate within the population as a whole. In addition, no studies have addressed CVS epidemiology among adults in Japan. The database encompassed 3.5 million active individuals, providing a large representative sample of the Japanese population with a substantial follow-up time. Furthermore, a symptom-based approach not only identified patients who had been diagnosed with CVS but also addressed the potential for underestimating the prevalence of the disease. Moreover, the usage status of therapeutic medicines can be confirmed, and data regarding usage status may indicate the degree of severity. For instance, patients who receive prophylactic therapy may experience a more severe form of the disease than those who can be managed without prophylactic treatment.

Our study has some limitations. Firstly, this symptom-based approach could have overestimated or underestimated the actual epidemiology of CVS in the Japanese population. CVS can be difficult to distinguish from gastroenteritis clinically, especially among children. We found that the concurrent diagnosis of gastroenteritis with vomiting was 93% for children, 74% for adolescents, and 31% for adults (data not shown). Since the limitation of the Rome IV criteria is the lack of specificity in children, the inclusion of gastroenteritis may very likely lead to overestimation bias. While gastroenteritis shares many similarities with CVS, we did not include patients diagnosed with gastroenteritis for the following reasons: Utilizing patients diagnosed with gastroenteritis would significantly affect the size of the pediatric CVS population. Without including gastroenteritis diagnoses in our investigation, the pediatric CVS estimation was similar to that in previous studies. However, we may have underestimated adult CVS, since it could be possible that an adult patient may have had multiple visits, which may have been labeled or mislabeled as gastroenteritis in a year. However, to avoid overestimation of CVS in the pediatric population, we did not include patients who had been previously diagnosed with gastroenteritis. We needed to apply this same limitation to adult patients to similarly evaluate each population, which may have caused an underestimation in adult patients with CVS. However, we included patients with gastroesophageal reflux disease (GERD) in this investigation. GERD is a common disease among adults, and this study included patients who presented with a history of GERD before vomiting for the following reasons. GERD was considered a comorbidity instead of a competing diagnosis. Moreover, sensitivity analysis showed that excluding patients with a history of GERD had a minimal impact on the results of the study. In effect, only 1% of cases were additionally excluded due to a concurrent GERD diagnosis. Lastly, this study identified patients with CVS by using the frequency with which they sought medical services for vomiting. Therefore, patients who relied on self-care at home via over-the-counter medications were excluded from the database. Ultimately, the invisibility of milder symptoms within the claims database, such as mild dyspepsia, may not have any significant impact on this study. This is supported by the fact that there is a universal health insurance program in Japan, and medical expenses for children are either free or affordable in many areas.

In summary, this study provides a key addition to the sparse epidemiological data on CVS in the literature. To the best of our knowledge, this is the first large population-based study to describe the incidence and prevalence of CVS across all ages in an Asian country, despite limitations. Epidemiological information projected that approximately 49,000 patients and 2,000–2,500 children and adolescents satisfy the Rome IV criteria for CVS annually across Japan. Furthermore, the study findings contribute to the knowledge related to patient characteristics and management as well as the low diagnosis of this disorder, especially among adults, in Japan. Further research is needed to fully understand the factors that may affect the outcomes and quality of life of patients with CVS. This study may shed light on the diseases that have been largely dismissed or unrecognized among Asian adults.

## Supporting information

S1 Table Definitions of medical conditions associated with vomiting. (TIF)
