# Supplementary figures and images for: Prevalence and incidence of cyclic vomiting syndrome in Japan: A study using Japanese claims data
Source: PLoS One. 2022 Dec 22;17(12):e0279502. doi: 10.1371/journal.pone.0279502 (PMC9778604; doi:10.1371/journal.pone.0279502)

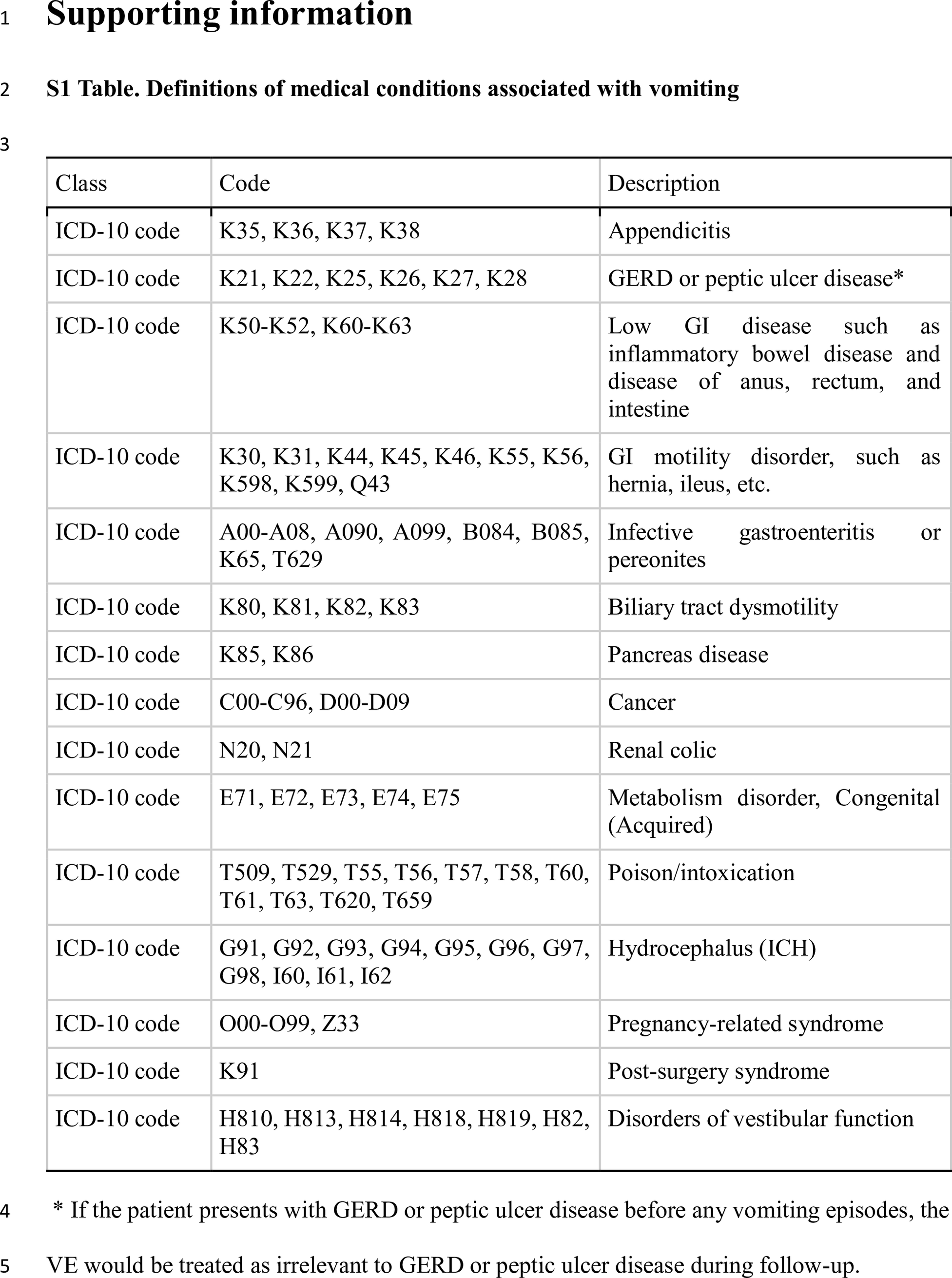

Supplement: S1 Table — (TIF) [file pone.0279502.s001.tif]
